# Genetic Manipulation Strategies for β-Thalassemia: A Review

**DOI:** 10.3389/fped.2022.901605

**Published:** 2022-06-15

**Authors:** Nur Atikah Zakaria, Rosnah Bahar, Wan Zaidah Abdullah, Abdul Aziz Mohamed Yusoff, Shaharum Shamsuddin, Ridhwan Abdul Wahab, Muhammad Farid Johan

**Affiliations:** ^1^Department of Haematology, School of Medical Sciences, Universiti Sains Malaysia, Kubang Kerian, Malaysia; ^2^Department of Neurosciences, School of Medical Sciences, Universiti Sains Malaysia, Kubang Kerian, Malaysia; ^3^School of Health Sciences, Universiti Sains Malaysia, Kubang Kerian, Malaysia; ^4^Institute for Research in Molecular Medicine (INFORMM), Universiti Sains Malaysia, Kubang Kerian, Malaysia; ^5^Universiti Sains Malaysia (USM)-RIKEN Interdisciplinary Collaboration for Advanced Sciences (URICAS), Penang, Malaysia; ^6^International Medical School, Management and Science University, Shah Alam, Malaysia

**Keywords:** β-thalassemia, splice-switching, antisense oligonucleotides, zinc finger nucleases, transcription activator-like effector nucleases, CRISPR-Cas9

## Abstract

Thalassemias are monogenic hematologic diseases that are classified as α- or β-thalassemia according to its quantitative abnormalities of adult α- or β-globin chains. β-thalassemia has widely spread throughout the world especially in Mediterranean countries, the Middle East, Central Asia, India, Southern China, and the Far East as well as countries along the north coast of Africa and in South America. The one and the only cure for β-thalassemia is allogenic hematopoietic stem cell transplantations (HSCT). Nevertheless, the difficulty to find matched donors has hindered the availability of this therapeutic option. Therefore, this present review explored the alternatives for β-thalassemia treatment such as RNA manipulation therapy, splice-switching, genome editing and generation of corrected induced pluripotent stem cells (iPSCs). Manipulation of β-globin RNA is mediated by antisense oligonucleotides (ASOs) or splice-switching oligonucleotides (SSOs), which redirect pre-mRNA splicing to significantly restore correct β-globin pre-mRNA splicing and gene product in cultured erythropoietic cells. Zinc finger nucleases (ZFNs), transcription activator-like effector nucleases (TALENs) and clustered regularly interspaced short palindromic repeats (CRISPR)/CRISPR-associated 9 (Cas9) are designer proteins that can alter the genome precisely by creating specific DNA double-strand breaks. The treatment of β-thalassemia patient-derived iPSCs with TALENs have been found to correct the β-globin gene mutations, implying that TALENs could be used as a therapy option for β-thalassemia. Additionally, CRISPR technologies using Cas9 have been used to fix mutations in the β-globin gene in cultured cells as well as induction of hereditary persistence of fetal hemoglobin (HPFH), and α-globin gene deletions have proposed a possible therapeutic option for β-thalassemia. Overall, the accumulated research evidence demonstrated the potential of ASOs-mediated aberrant splicing correction of β-thalassemia mutations and the advancements of genome therapy approaches using ZFNs, TALENs, and CRISPR/Cas9 that provided insights in finding the permanent cure of β-thalassemia.

## Introduction

Thalassemia, an autosomal recessive hematologic disease is becoming a serious health problem worldwide with its high prevalence and incidence (1). Thalassemia is a condition in which one of the genes that code for the adult hemoglobin components, the α-and β-globin chains, are altered or missing. The globin genes can be affected by a variety of mutations, resulting in thalassemia. The β-globin gene (HBB), which exclusively encodes for β-globin chains, is reduced or missing in β-thalassemia. β-thalassemia is prevalent in Mediterranean countries, the Middle East, Central Asia, India, Southern China, and the Far East as well as countries along the north coast of Africa and in South America. Around 1.5 percent of the world's population (80 to 90 million people) is estimated to be β-thalassemia carriers, with a yearly incidence of symptomatic β-thalassemia individuals of 1 in 100,000 people globally (2).

The severity of β-thalassemia is described as three main forms: thalassemia major, thalassemia intermedia and thalassemia minor. Thalassemia major presents with severe anemia that necessitates regular red blood cell (RBC) transfusions within the first 2 years of life. Patients with untreated or inadequately transfused thalassemia major commonly experience growth retardation, pallor, jaundice, weak musculature, hepatosplenomegaly, leg ulcers, extramedullary hematopoiesis, and skeletal abnormalities as a result of bone marrow expansion. On the other hand, blood transfusions on a regular basis might lead to an excess of iron in the blood. Excess iron can cause serious and irreversible organic damage, such as cirrhosis, diabetes, heart disease, and hypogonadism, if it is not well-managed (3). The most common cause of death of β-thalassemia is secondary to cardiovascular diseases due to either severe anemia (before the era of regular blood transfusions) or iron overload (after the implementation of transfusion therapy), followed by infection (4–6).

Patients with thalassemia intermedia present later in life with moderate anemia and do not require regular transfusions. Main clinical features in these patients are hypertrophy of erythroid marrow with medullary and extramedullary hematopoiesis and its complications (osteoporosis, masses of erythropoietic tissue that primarily affect the spleen, liver, lymph nodes, chest and spine, and bone deformities and typical facial changes), gallstones, painful leg ulcers and increased predisposition to thrombosis (7). Thalassemia minor is clinically asymptomatic but some subjects may have moderate anemia with symptoms such as headache, lethargy, fatigue, dizziness, and exercise intolerance (8).

Currently, there is no other curative option for β-thalassemia except allogenic hematopoietic stem cell transplantation (HSCT). Nevertheless, this therapeutic option is not widely available for β-thalassemia patients due to the difficulty to find matched human leukocyte antigen (HLA) bone marrow donors. HSCT of mismatched HLA donor could lead to transplantation failure due to severe graft vs. host disease (GVHD) and treatment-related death (9, 10). Clinically, some studies have reported the outcomes of allogenic HSCTs application on β-thalassemia patients. A retrospective review was conducted to evaluate the clinical outcomes of allogenic HSCTs children with β-thalassemia major in a Jordan health center. Out of 34 patients included in this study, the overall survival was 97% and thalassemia free survival was 88.2% (11). In one of the health centers in Italy, a study was conducted on 80 β-thalassemia patients that received allogenic HSCTs. A total of 93.7% of patients being thalassemia-free, proving the benefit of HSCTs for most patients. Allogenic HSCT is still associated with GVHD (12.7%), graft failure (10%), and mortality (3.8%) (12). The autologous HSCT approach primarily serves as a promising therapy to cure β-thalassemia, however, there are some major challenges such as controlling transgene expression, which ideally should be erythroid-specific, differentiation stage-restricted, elevated, position independent, and sustained over time (13).

The availability of new tools and techniques in recent years has accelerated the development of gene-editing treatments to ameliorate the pathophysiology in β-thalassemia patients. In this review, we provide an overview of the genome therapeutic approaches for the β-thalassemia, including RNA manipulation therapy, splice-switching, gene editing and generation of corrected induced pluripotent stem cells (iPSCs).

## Molecular Basis of β-Thalassemia

The quantitative reduction in β-globin production depends on the underlying molecular mechanisms. HBB mutations that result in no β-globin production leads to β^0^-thalassemia. Other mutations that impair the β-globin synthesis at a variable degree are classified as β^+^- or β^++^- (“silent”) thalassemia. The quantitative reduction of β-globin causes its hemoglobin tetramer partner, α-globin to be in excess. The accumulation of free α-globin is responsible for the pathophysiology of β-thalassemia by which the degree of imbalance between α- and non-α-globin chain synthesis influences the severity of the β-thalassemia phenotype.

Some structurally abnormal β-globin variants are also quantitatively reduced, with a phenotype of β-thalassemia. To date, there are more than 1,400 hemoglobin variants that have been reported, with more than 900 variants in HBB gene (14). The most common β-globin structural variants are hemoglobin E (HbE [β26 Glu>Lys]), sickle hemoglobin (HbS [β6 Glu>Val]), and hemoglobin C (HbC [β6 Glu>Lys]) (15).

**Figure 1:**
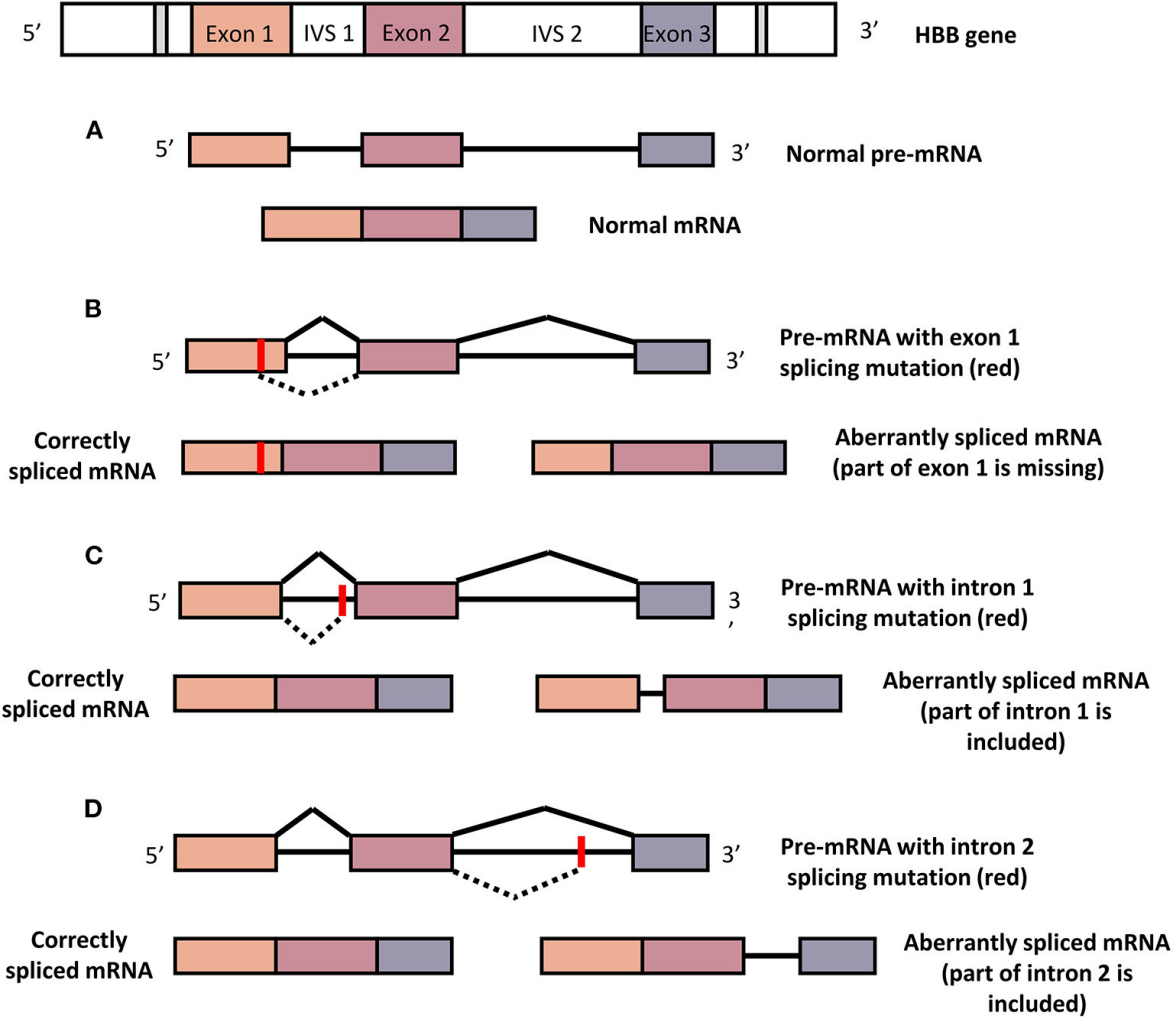
Normal and aberrant splicing mechanisms. **(A)** Normal HBB produce normal pre-mRNA with intact three exons and subsequently translated into normal β-globin. **(B)** HBB with mutation in exon 1 that activates a *de novo* splice site may either produce correctly spliced or aberrantly spliced mRNA which later be translated into normal or no β-globin, respectively. **(C)** HBB with intron 1 mutation may activate correct or aberrant splicing pathways that give rise to normal or no β-globin, respectively. **(D)** Intron 2 mutation in HBB may induce correct or aberrant splicing mechanisms that yield normal or no β-globin, respectively. Red point marked the mutation location. Dashed line indicates the aberrant splicing mechanisms.

β^E^ (CD26) is one of the most common β-thalassemic mutations among Southeast Asians that affect β-globin pre-mRNA splicing, thus producing abnormal hemoglobin, HbE (16). During the translation of the gene into protein, introns in the pre-mRNA need to be removed through the splicing process (Figure 1). HBB mutations at the splice junctions activates the aberrant splice sites that reduces the efficiency of the normal splicing pathway, leading to the non-functional β-globin chain production that give rise to β-thalassemia. Other HBB aberrant splicing mutations are CD19 and CD27 mutations in exon 1, IVS1-5, IVS1-6, and IVS1-110 mutations in intron 1, IVS2-654, IVS2-705 and IVS2-745 mutations in the intron 2 (17). HBB carrying the cryptic splice site on coding sequence (exons) may either be translated into a β-globin variant (eg: β^E^-globin, Hb Malay) or into a defective mRNA that will later be degraded in the nucleus of the cells.

Almost 50% of the severe β-thalassemia patients worldwide were found with the underlying double heterozygosity for a β-thalassemia mutation and the β^E^ mutation (18). HbE/β-thalassemia has a highly varied phenotype, with many patients remaining mostly transfusion-free throughout their lives, while others are initiated on transfusion at an early age (19).

## RNA Manipulation Strategies

Occurrence of aberrant splicing is one of the processes that affects β-globin synthesis in β-thalassemia even though the correct splice sites remain potentially functional. By blocking the aberrant splice sites or other sequence elements related to splicing with antisense oligonucleotides, the splicing machinery may be forced to reselect the right splice sites and drive the synthesis of β-globin mRNA and polypeptide, therefore restoring gene function.

### Antisense Oligonucleotides

**Figure 2:**
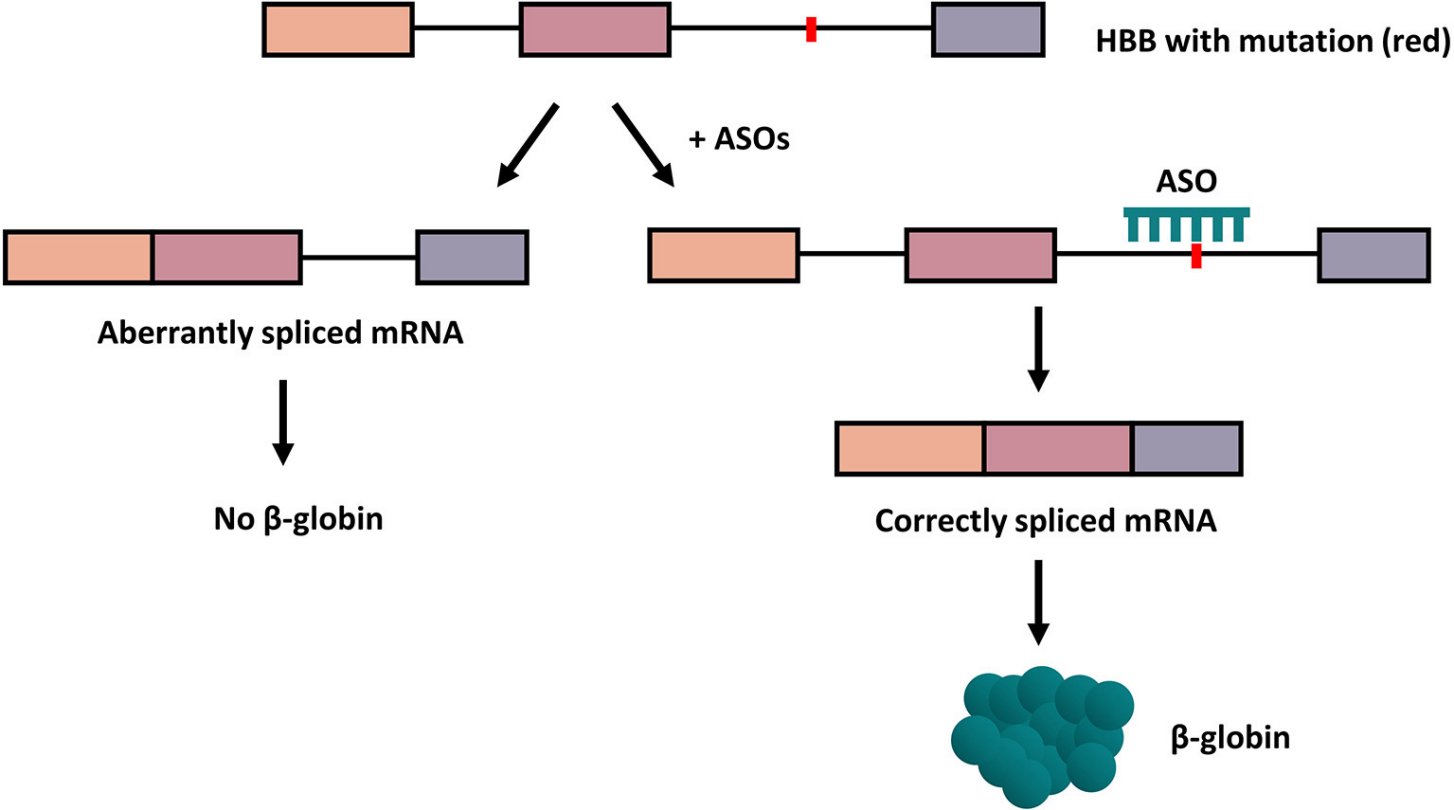
How ASOs work to correct the aberrant splicing. In the presence of ASOs targeted at the mutation on the gene, the correction of aberrant splicing of human β-globin occurs and leads to the production of functional β-globin protein. Boxes indicate exons; lines, introns; red dots, mutation.

Antisense oligonucleotides (ASOs) are non-ionic DNA analogs possessing altered backbone linkages relative DNA or RNA that bind complementarily to nucleic acid sequences by Watson-Crick base-pairing (20). ASOs include antisense morpholino oligonucleotides (AMOs) and splice-switching oligonucleotides (SSOs). To improve the pathophysiology of β-thalassemia, the ASOs entered the erythroid progenitor cells, migrated to the nucleus, and hybridized to the aberrant splice sites to suppressed the aberrant splicing pattern of β-globin pre-mRNA. In consequence, the correct splicing was restored and increased the expression of functional β-globin (Figure 2).

Aberrant splicing mutations in intron 2 of the HBB include mutations at nucleotides 654, 705, or 745 (β^IVS2−654^, β^IVS2−705^ and β^IVS2−745^-globin). A cytosine to thymine mutation at nucleotide 654 of human β-globin intron 2 (β^IVS2−654^) is one of the most common mutations causing β-thalassemia in Chinese and Southeast Asians that affect β-globin pre-mRNA splicing (21). In a study, erythroid progenitor cells derived from β-thalassemia major (IVS2–745/IVS2–745 and IVS2–745/IVS2–1) and β-thalassemia intermedia (IVS2–654/β^E^) were treated with AMOs which targeting at the aberrant splice sites in the β-globin gene. This results in efficient restoration of correctly-spliced β-globin pre-mRNA and subsequently increase the level of hemoglobin A (HbA), suggesting the potential clinical application of AMOs to correct the aberrant splicing of β-thalassemia (22).

Furthermore, free uptake of AMOs in transgenic mice harboring IVS2-654 HBB has resulted in restoration of correct human β-globin mRNA in the erythroid cells of the transgenic mice. The effects of free uptake of AMOs were also tested on erythroid precursor cells derived from IVS2–654/β^E^-thalassemia patients and as a response, increased levels of β-globin mRNA and hemoglobin A was recorded. Thus, these findings indicate the potential of restoring the β-globin mRNA splicing via free uptake of AMOs (23).

In another experiment, IVS2-654 in intron 2 of HBB was repaired in a study by Svasti and her colleague using an *in vivo* mouse model of IVS2–654 thalassemia. The aberrant splice site in the pre-mRNA was targeted using SSOs. Significant amounts of hemoglobin were restored in the peripheral blood of the IVS2-654 mouse, suggesting a promising alternative to correct the aberrant splicing of IVS2-654 mutation in β-globin (24).

HeLa cells that are stably expressing β^E^/IVS1-6 were established by Suwanmanee et al. (25). They utilized this cell line as a tool to correct the aberrant splicing of β^E^/IVS1-6 by using AMOs. Interestingly, the treatment of AMOs increased the amount of correctly spliced β^E^-globin mRNA in a dose-dependent and sequence-specific manner. It was quite promising when application of the same AMOs to erythroid progenitor cells from two HbE/β-thalassemia increased the production of β^E^-globin mRNA and HbE by 70 and 36%, respectively (25).

To support the previous findings, El-Beshlawy et al. (26) studied *ex vivo* correction of aberrant β^IVS1−110^-globin pre-mRNA splicing by ASOs. A total of 10 peripheral blood mononuclear cells were derived from 10 β^IVS1−110^ thalassemia patients. Fifty percent of the cases showed correction of the aberrant splicing; 2 of them showed corrected mRNA band with no aberrant mRNA band while another 3 showed increased ratio between corrected to aberrant mRNA band. In addition, significant increase of total hemoglobin level was also observed in those five corrected cases, suggesting that antisense oligomers are applicable for treating β-thalassemia (26).

Another aberrant splicing mutation of β-thalassemia, IVS2-745 (C>G) located in intron 2 of HBB, causes a premature in-frame termination codon that inhibits β-globin production. The aberrant splicing in the pre-mRNA was reversed using uniform 2'-O-methoxyethyl (2'-MOE) SSOs resulting in up to 80% increase of adult hemoglobin in erythroid cells of β^IVS2−745^-thalassemia patients. Moreover, the balance between β-like and α-globin chains was restored, thus leading to reduction of toxic heme aggregates up to 87%. These findings suggest the potential application of 2'-MOE SSOs to restore the aberrant splicing in β-thalassemia patients in future (27).

Successful repair of β-globin pre-mRNA splicing defect by synthetic ASOs in erythroid cells was demonstrated. The advantages of antisense treatment include; (1) ASOs correct splicing of pre-mRNA that is transcribed from the native β-globin locus thus precluding overexpression of β-globin mRNA, (2) the approach offers a pharmacological treatment, easier to implement than gene therapy, and (3) the treatment may be easily stopped if any undesirable effects are observed (22). ASOs, however, possess inherent drawbacks. Its clinical application is limited by short-term effectiveness and the requirement for lifelong periodic administration of the ASOs to maintain therapeutic levels of β-globin (21). Moreover, this approach is not applicable to β-thalassemia with other genotypes and is limited in comparison to gene therapy, which may replace or supplant any mutant with a correct gene (22).

### U7 snRNA

**Figure 3:**
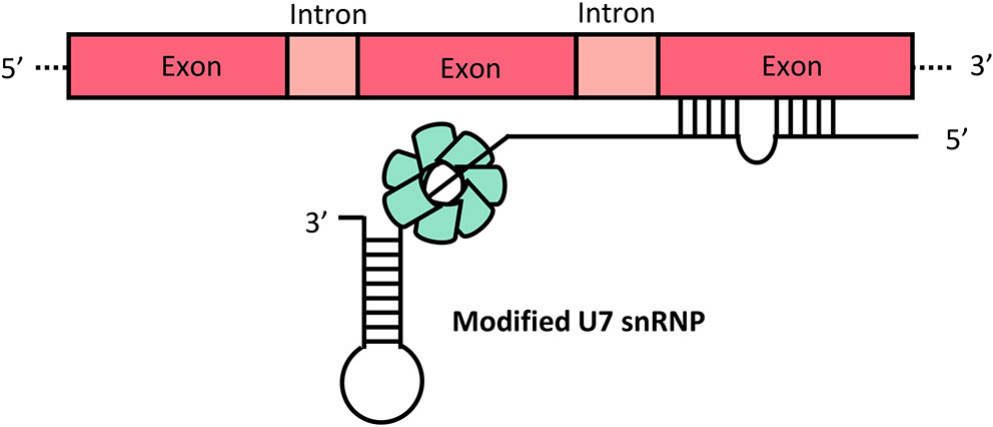
Modified U7 snRNP. Complexes of spliceosome proteins (green) combine with U7 snRNA that is incorporated with antisense oligonucleotides targeted to the aberrant splice site of the gene. Boxes represent exons and introns.

Engineering of viral vector mediated expression of U7 snRNA carrying the ASOs that restores the correct splicing of HBB potentially overcomes the short-term effectiveness of ASOs treatment. Small nuclear RNAs that are rich in uridine are known as U snRNAs and are numbered in order of discovery. The spliceosomes, large complexes that catalyze splicing, are divided into major and minor spliceosomes: U1, U2, U4, U5, U6 and U11, U12, U4atac, U5atac and U6atac snRNP, respectively. Although the U7 snRNP is not involved in splicing, it is a critical element in the processing of replication-dependent histone (RDH) pre-mRNAs at their unique 3' end. Furthermore, findings based on modified U7 snRNP (U7 Sm OPT) targeting splicing to induce efficient skipping or inclusion of specified exons have demonstrated U7snRNA as a useful tool in therapeutic trials. In U7 Sm OPT-based therapy, an antisense oligonucleotide is incorporated into the U7 snRNA, forming modified U7 small nuclear ribonucleoproteins (U7 snRNP) (Figure 3) (28).

Vacek et al. (29) transfected the HeLa cells expressing the mutations at nucleotides 654, 705 and 745 in intron 2 of HBB with modified U7 snRNA (U7.623) to prevent the aberrant splicing activated by the mutations. U7.623 that contains antisense sequence was able to reduce the incorrect splicing of the β-globin pre-mRNA and increase the level of correctly-spliced β-globin mRNA. Application of U7.623 in hematopoietic stem cells and erythroid progenitor cells derived from IVS2-745/IVS2-1 β-thalassemia patients resulted in approximately 25-fold increase in the levels of correctly-spliced β-globin mRNA and hemoglobin A. These findings proved the lentiviral vector-based gene therapy for β-thalassemia (29).

A modified U7 snRNA targeting the IVS2-654 β-globin pre-mRNA was delivered by lentivirus into iPSCs derived from mesenchymal stromal cells of a patient with HbE/β^IVS2−654^-thalassemia. The efficiency of the modified U7 snRNA was proven with the high level of correctly spliced β-globin mRNA in erythroblasts differentiated from the transduced iPSCs. The modified U7 snRNA has great potential to provide the autologous iPSCs transplantation to restore the aberrant splicing of β-thalassemia (30).

An engineered U7 snRNA targeted to several pre-mRNA splicing elements on the β^IVS2−654^-globin pre-mRNA, U7.BP + 623, was effective in a HeLa cell line carrying the IVS2-654 by which the correctly-spliced β^IVS2−654^-globin mRNA was increased. Progenitor cells derived from HbE/β^IVS2−654^-thalassemia patients were transduced with lentiviral-mediated U7.BP + 623 and promoted restoration of correct splicing of β^IVS2−654^-globin mRNA as well as restoration of HbA production. This finding marked the potential usage of the lentiviral-mediated engineered U7 snRNA as an alternative for the long-term treatment of β-thalassemia (21).

A following research was performed to evaluate the effect of U7.BP+623 on β^IVS2−654^-globin pre-mRNA splicing in β^IVS2−654^-thalassemia mice erythroid progenitor cells. As expected, the correction of β-globin pre-mRNA splicing was achieved. However, the level of correctly spliced β-globin was lower than previously reported in patient. This situation was probably due to an inefficient processing of U7 snRNA in mouse thus producing truncated engineered U7 snRNA. The different processing of U7 snRNA in humans and mice has therefore restricted the depth analysis of the engineered U7 snRNA in the mouse model (31).

In an attempt to correct the aberrant splicing of CD26 mutation, Preedagasamzin et al. (32) proved that the U7 bE4þ1 snRNA lentiviral vector was effective in restoring the correctly-spliced β^E^-globin mRNA for at least 5 months. Application of the same lentiviral vector in erythroid progenitor cells from HbE/β-thalassemia patients also resulted in the increase of the correctly-spliced β^E^-globin mRNA thus suggesting the long-term treatment for the HbE/β-thalassemia patients using the engineered U7 snRNA lentiviral vector (32).

The strategy of the engineered U7 snRNA that is reported here may be a new alternative approach for β-thalassemia gene therapy, even though improvements of the vector system are still required (21). Additionally, the engineered U7 snRNA mediated splicing correction can be implemented in numerous other diseases caused by RNA mis-splicing (32).

## Genome Manipulation Strategies

Recent advancements that permitted seamless engineering of the human genome using variety of technologies are collectively referred to as gene editing. Precision gene editing technologies enable the alteration of the genome at precise loci, resulting in targeted genomic changes that are being used in a variety of medical applications. The clustered regularly interspaced short palindromic repeats (CRISPR)/CRISPR-associated 9 (Cas9) system is a widely used tool for genome engineering, however it is not the first of its kind. Zinc-finger nucleases (ZFNs) and transcription activator–like effector nucleases (TALENs), for example, are programmable protein-based genome engineering tools that have been widely used. In terms of design and cost, ZFNs are challenging. To test the cutting efficiency of TALENs, many pairs must be made. Furthermore, DNA methylation and histone acetylation may have an impact on their effectiveness. CRISPR/Cas9, on the other hand, is not limited by these constraints, is practical, and is simple to manufacture. The major goals of gene therapy are to transfer a healthy copy of HBB or to re-establish the expression of γ-globin, and hence fetal hemoglobin (HbF) (33, 34).

**Figure 4:**
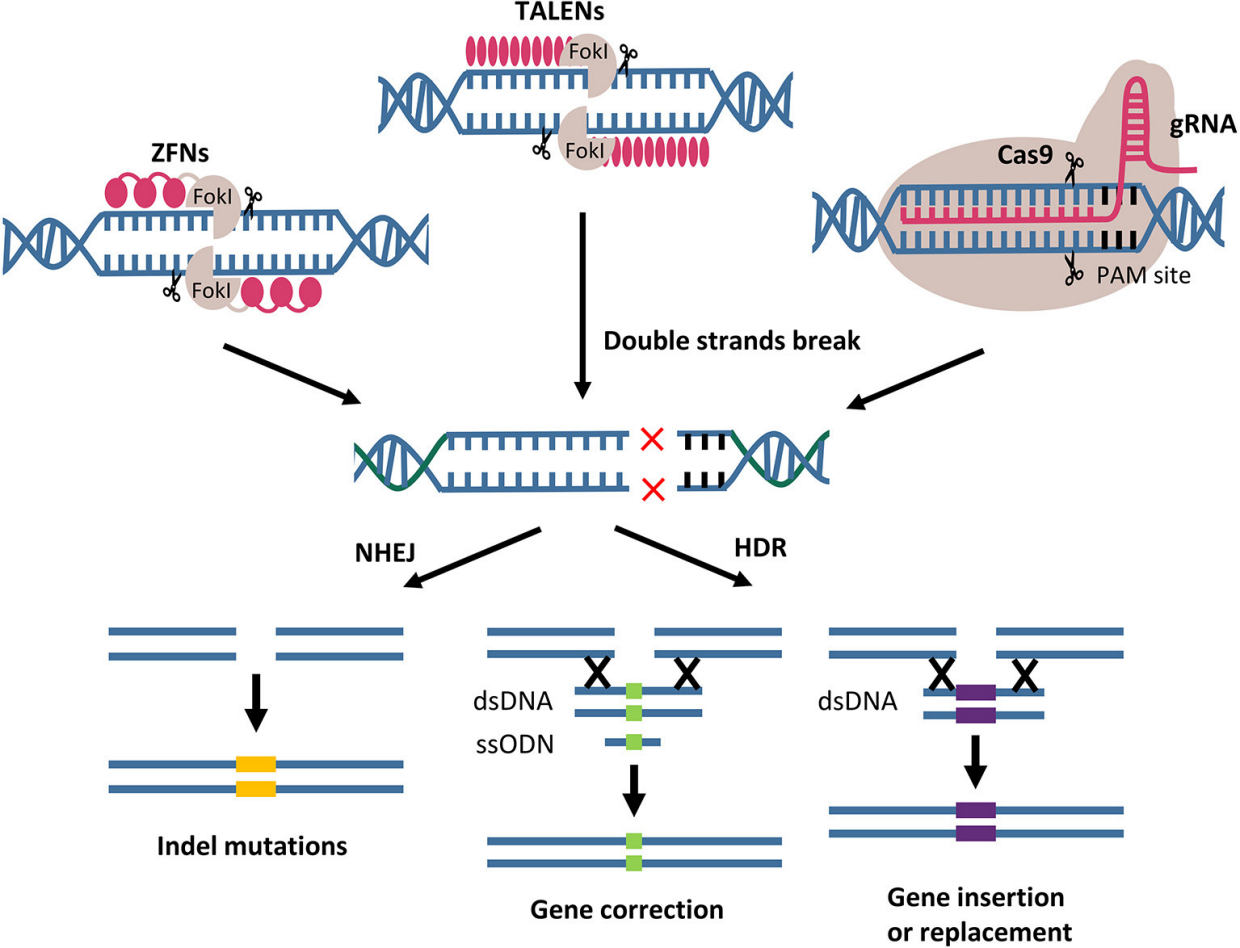
Gene editing by designer nucleases. ZFNs, TALENs, and CRISPR/Cas9 mediated the genome modifications through two main double strand break repair pathways. Indel mutations resulted from NHEJ pathway. Gene correction, insertion and replacement using DNA donor template are the outcomes of HDR pathway. FokI, endonuclease from *Flavobacterium okeanokoites*; PAMs, protospacer adjacent motifs; NHEJ, non-homologous end joining; HDR, homology-directed repair; dsDNA, double-stranded DNA; ssODN, single strand oligodeoxynucleotides.

Genome editing using designer nucleases results in the formation of DNA double-strand breaks (DSBs) at particular genomic loci. DSBs can be repaired in the cell via homology-directed repair (HDR) and non-homologous end joining (NHEJ). HDR is a high-fidelity repair pathway that permits a homologous DNA donor template to be integrated at a particular location and potentially being exploited to correct disease-causing mutations. The error-prone NHEJ mechanism has mostly been used to generate minor insertions and deletions in order to achieve permanent gene inactivation and disruption of gene expression (Figure 4).

### ZFNs and TALENs

ZFNs are composed of three to six zinc-fingers that bind DNA sequences and a cleavage domain, FokI, to generate DSBs. In the last decade, studies have demonstrated efficient targeted integration in HSPCs by ZFNs expression with exogenous homologous recombination donors delivered via single-stranded oligonucleotides (35), integrase-defective lentiviral vectors (36), or recombinant adeno-associated viral vectors of serotype 6 (rAAV6) (37, 38). Nevertheless, research on the use of ZFNs in editing HBB gene is limited due to challenges in developing the ZFNs. As FokI-dependent DNA cleavage requires dimerization of the cleavage domains, two ZFNs are required for binding DNA (on both strands and in opposite direction) to align FokI domains and allow DNA cleavage (39). Furthermore, laboratory procedures are time-consuming without a ZFNs specialist, resulting in a large amount of effort being required to generate a successful edit. One of the key advantages of the ZFN system is the utilization of dimerized FokI, which can improve the specificity of DNA targeting while simultaneously reducing off target effects. Notably, a developed ZFN is already in the clinical trial stage, ST-400 that targets *BCL11A*, a master regulator of the fetal-to-adult hemoglobin switch (NCT03432364, https://clinicaltrials.gov/). Detailed informations of ZFN-mediated clinical trials are listed in Table 1. The preliminary results of a phase 1/2 clinical study in transfusion-dependent β-thalassemia patients showed a relatively low genome editing efficiency associated with poor HbF expression (40).

**Table 1:** Clinical trials using the ZFN-mediated technology (https://clinicaltrials.gov/).

**Status**	**Study title**	**Conditions**	**Intervention**	**Locations**	**Informations**
Recruiting	An observational long-term safety and efficacy follow-up study after ex-vivo gene therapy with bivv003 in severe sickle cell disease (SCD) and St-400 in transfusion-dependent beta-thalassemia (TDT) with autologous hematopoietic stem cell transplant (NCT05145062)	Blood and lymphatic diseases	ST-400	Detroit, Michigan, United States	This study evaluates long-term safety of BIVV003 in participants with severe sickle cell disease (SCD) and ST- 400 in participants with transfusion-dependent beta-thalassemia (TDT)
Active, not recruiting	A study to assess the safety, tolerability, and efficacy of ST-400 for treatment of transfusion-dependent beta-thalassemia (TDT) (NCT03432364)	Transfusion dependent beta-thalassemia	ST-400 investigational product	University of California, Los Angeles, California, United States UCSF Benioff Children's Hospital, Oakland, California, United States Children's Healthcare of Atlanta, Atlanta, Georgia, United States (and 3 more...)	ST-400 uses ZFN technology to disrupt a precise and specific sequence of the enhancer of the BCL11A gene (which normally suppresses fetal hemoglobin production in erythrocytes). This process is intended to boost fetal hemoglobin (HbF), which can substitute for reduced or absent adult (defective) hemoglobin.

TALENs are chimeric proteins that contain two functional domains: a DNA-recognition transcription activator-like effector (TALE) and a nuclease domain. TALENs serves as customizable restriction enzymes that recognize a specific sequence and introduce an overhang double-stranded break. TALENs are considerably easier to design compared to ZFNs, thus explaining their widespread use.

Ma et al. (41) combined the integration-free β-thalassemia induced pluripotent stem cells (iPSCs) derived from patients and TALEN-based universal correction of β-globin mutations *in situ*. This robust process has successfully corrected the HBB and the corrected iPSCs can be induced to differentiate into hematopoietic progenitor cells and then further to erythroblasts expressing normal β-globin. This finding suggests the efficient and promising strategy to correct different types of β-globin mutations in β-thalassemia iPSCs (41).

TALENs were also being used to investigate the efficiency of correcting the aberrant splice sites in homozygous erythroblast derived from IVS1-110(G>A)-homozygous patient. As a result, significant correction at RNA, protein and morphological levels were observed, suggesting the disruption of aberrant regulatory elements by TALENs as a highly efficient gene therapy approach for suitable mutations (42).

In a study to compare between TALENs and CRISPR/Cas9, Xu et al. (43) designed both endonucleases targeting at IVS2-654 mutation in β-globin of patient-derived iPSCs. Different frequencies of double-strand breaks (DSBs) at IVS2-654 were observed when using TALENs and CRISPR/Cas9 in which TALENs have higher gene targeting efficiency. Further differentiation of TALENs-corrected iPSCs clones generated a higher transcription of β-globin compared to the uncorrected cells. These findings may guide the future application of TALENs in the treatment of β-thalassemia and other monogenic diseases (43).

In depth analysis of TALENs-mediated NHEJ correction of β-thalassemia mutation was performed in mice model carrying a IVS2-654 mutation on the β-globin gene. TALENs vectors targeting at the mutation were constructed and used to generate mice with TALENs^+^/β^IVS2−654^ genotype. The sequencing analysis revealed that the IVS2-654 mutation was deleted in 50% of TALENs^+^/β^IVS2−654^ mice and no off-target effects were observed. Western blot analysis confirmed the expression of normal β-globin. There are decreases in proportion of nucleated cells in the bone marrow, splenomegaly with extramedullary hematopoiesis, and iron deposition in the spleen and liver of TALENs^+^/β^IVS2−654^ mice. These findings suggest a straightforward strategy to treat anemia in β-thalassemia (44).

ZFNs and TALENs have been shown to edit numerous loci in multiple cell types. While TALENs are frequently linked with less cytotoxicity than ZFNs, their larger size may constrain their delivery to therapeutically important cells for gene therapy applications. Both ZFNs and TALENs have the potential to cause off-target effects that may be detrimental to the target cells. Finally, their complex design may hinder their development for genome editing procedures (39).

### CRISPR/Cas9 and Cas12a

The CRISPR/Cas9 technology was first introduced in 2012 and has resulted in a paradigm shift in the field of genome editing. The major difference between this system and other nucleases (i.e. ZFNs and TALENs) is that the Cas9 nuclease is directed to the DNA by Watson-Crick base pairing rather than a protein-DNA interaction via an RNA molecule (guide RNA, gRNA). Cas9 activity is dependent on the presence of protospacer adjacent motifs (PAMs), which are short sequences downstream of the target sequence (protospacer) that are complementary to the gRNA. PAMs are unique to the Cas9 generated from each bacterial species.

β-thalassemia iPSCs that had a CD17 (A>T) homozygous point mutation in HBB was corrected with CRISPR/Cas9 producing gene-corrected iPSCs that possess normal karyotype and maintained pluripotency without any off-targeting effects. The differentiation efficiency was evaluated and proven by the increased embryoid body ratio and various hematopoietic progenitor cells percentages. Notably, the gene-corrected β-thalassemia iPSCs restored HBB expression and reduced reactive oxygen species production compared to un-corrected group, implying that CRISPR/Cas9 system had greatly improve the hematopoietic differentiation efficiency of β-thalassemia iPSCs (45).

Xiong et al. (46) studied the combination effect of CRISPR/Cas9 and single strand oligodeoxynucleotides (ssODNs) in iPSCs derived from IVS2-654 β-thalassemia patients. CRISPR/Cas9 is targeted at the IVS2-654 mutation site on HBB and mediates the double strand breaks (DSBs). ssODNs are then seamlessly corrects the gene. The corrected iPSCs maintained the pluripotency and are able to differentiate normally, thus producing correct β-globin. The strategy of combining CRISPR/Cas9 system and ssODNs provides the promising gene correction of β-thalassemia and can be considered as future approaches for management of β-thalassemia (46).

Xu et al. (47) demonstrated the disruption of the aberrant splice site targeted at IVS1-110G>A mutation using Cas9 ribonucleoprotein (RNP) and IVS2-654C>T mutation by Cas12a/Cpf1 RNP in primary CD34+ hematopoietic stem and progenitor cells (HSPCs) from β-thalassemia patients. In regards with the high efficiency and penetration of Cas9 and Cas12a, the edited patient HSPCs showed reversal of aberrant splicing and restoration of β-globin expression. Notably, up to 73% InDel was observed and frequent 1-bp insertions at the IVS1-110 site was enough to restore normal β-globin splicing. The application of this gene editing technology offers a bright future for the treatment of transfusion-dependent β-thalassemia genotypes (47).

CRISPR/Cas9 gene editing technology was also being applied in CD34+ cells from Egyptian β-thalassemia patients with a IVS1-110 mutation. Cas9 and guide RNA were transfected into the CD34+ cells and causing DSBs at the target site and knocked out the IVS1-110 mutation. The corrected CD34+ cells gained the wild-type HBB and then were subjected for differentiation by culturing them in complete media containing erythropoietin. This study supported the existing studies for the application of CRISPR/Cas9 to treat β-thalassemia (48).

A strategy for correcting the −28 (A>G) and the 4-bp (TCTT) deletion at codons 41 and 42 in exon 2 was developed by reprogramming the patient-derived iPSCs through combination of CRISPR/Cas9 technology and the piggyBac transposon. A seamless correction of the HBB mutations through the HDR-based strategy was achieved with no off-target effects. On top of that, the cells also able to maintain their full pluripotency and exhibit normal karyotypes. After differentiation of the corrected iPSCs into erythroblasts, the gene-corrected iPSCs were successfully restored the expression of HBB compared to the parental iPSCs line. The seamless HBB correction demonstrating a critical step toward the stem cell-based gene therapy in the future (49).

Another efficient technique to correct −28(A>G) mutation was also developed by combining CRISPR/Cas9 with asymmetric single-stranded oligodeoxynucleotides (assODNs). Using K562 cell line carrying −28(A>G) mutation, the transcriptome level was compared with K562 cell line and found that the mutation disturbed the transcription and expression of β- and γ-globin gene. Interestingly, the abnormalities due to the −28(A>G) mutation were corrected by CRISPR/Cas9 and asymmetric assODNs in K562^−28(*A*>*G*)^. This study is the first to report on the whole-transcriptome analysis based on isogenic cell lines, thus it provides a platform to conduct future investigation of the mechanism of −28(A>G) β-thalassemia (50).

In two different groups, Liu and Niu applied CRISPR/Cas9-mediated HDR-based approaches to the iPSCs derived from β-thalassemia patients carrying 4-bp deletion (–TCTT) and (–CTTT) at CD41/42 mutation on β-globin gene respectively (51, 52). Specific CRISPR/Cas9 to target the mutation site was designed and they combined it with ssODNs. The repaired cells expressed normal β-globin transcripts when developed into hematopoietic progenitor cells and later erythroblasts. Notably, the corrected cells had a low mutational load and no off-target mutagenesis, as revealed by off-target analysis and whole-exome sequencing (52). These researches demonstrate the most efficient and safe method for genetically correcting the CD41/42 4-bp deletion in iPSCs through CRISPR/Cas9 and ssODNs to cure monogenic disease-associated mutations in patient-specific iPSCs.

The G>A point mutation in codon 26 of HBB was also corrected using guide RNA, Cas9 and ssODNs donor template via HDR-based approach. iPSCs was generated from human dermal fibroblasts of HbE/β-thalassemia patient's carrying CD41/42 mutation in one allele, and CD26 mutation in the other. After hematopoietic differentiation, the restoration of HBB protein expression was observed indicating that a single allele genetic correction of CD26 is sufficient to normalize the β-globin level in HbE/β-thalassemia. Nevertheless, only 2.9% of iPSCs clones showed efficient gene correction owing to poor transfection and HDR efficiency. The HDR efficiency might be improved by suppressing the NHEJ pathway once, however, the theory needs to be properly assessed in the future study (53).

Cosenza et al. (54) aimed to correct one of the most frequent β-thalassemia in Mediterranean area, β^0^39-thalassemia mutation, by utilizing the advanced technology of CRISPR/Cas9 gene editing. After CRISPR/Cas9 gene correction on erythroid precursor cells obtained from homozygous β^0^39-thalassemia patients, they demonstrated the presence of normal β-globin genes with high amount of normal β-globin mRNA and protein. Subsequently, the HbA level increased significantly and therefore reduce the excess free α-globin. These findings promote the development of this technique for the β^0^39-thalassemia patients and the positive outcomes may be maximized through the use of other therapeutic approaches such as reactivation of HbF (54).

Interestingly, Cai and coworkers established a novel universal approach that could potentially cure various β-thalassemia-causing mutations. This strategy is based on targeted integration of a donor template containing the complementary DNA (cDNA) that encodes the wild-type HBB gene. β-globin production was restored in erythrocytes derived from iPSCs of two transfusion-dependent β-thalassemia patients HBB that carry mutations CD17/IVS2-654 and CD17/CD41/42. This strategy of restoring functional HBB gene expression is expected to be clinically effective for permanently curing β-thalassemia patients with various HBB mutations in the future (55).

Coexistent of hereditary persistence of fetal hemoglobin (HPFH) and β-thalassemia have been long known to minimize the hematological abnormalities and result in a mild clinical manifestation. HPFH is a condition with consistently high HbF production that present in adulthood, which usually due to mutations in the β- or α-globin gene cluster or the γ promoter gene region. Ye et al. (56) mimics the HPFH genotype in bone marrow-derived adult CD34+ HSPCs by delivering target site-specific SaCas9 to remove part of β-globin locus and repaired by non-homologous end joining (NHEJ). Up to 31% of CD34+ HSPCs were successfully edited with the 13-kb HPFH5 deletion and after differentiation into erythroid cells *in vitro*, these cells significantly expressed the γ-globin compared to cells without HPFH deletions. Therefore, this study proved the potential new approach to autologous transplantation therapy to treat homozygous β-thalassemia (56).

One of the factors that determine the severity of β-thalassemia is the number of α-globin genes (*HBA1* and *HBA2*) by which α-globin gene deletions ameliorate β-thalassemia through the balanced ratio of α- and β-globin. A novel strategy was developed by combining 2 therapeutic approaches mediated by CRISPR/Cas9; deletion of HBA2 gene to recreate α-thalassemia trait with reduced α-globin production and integration of β-globin transgene downstream the *HBA2* promoter to increase β-globin expression. The study demonstrated the correction of the α/β-globin imbalance in erythroblast derived from edited β-thalassemia HSPCs, suggesting a novel therapeutic strategy for the treatment of β-thalassemia in the future (57).

CRISPR-Cas9 system serves some major advantages including: (1) ease of design and cloning, (2) high genome editing efficiency, (3) low cytotoxicity and transient expression when delivered as ribonucleoproteins (RNPs) versus mRNA delivery (which is typically used for ZFNs and TALENs), and (4) the possibility of multiplexing, which enables simultaneous targeting of multiple loci (58). Taken together, these benefits make CRISPR-Cas technology the optimal tool for developing therapeutic methods. To date, a number of clinical trials have been recorded to evaluate the potential of CRISPR-mediated technology in clinical settings as listed in Table 2.

**Table 2:** Clinical trials using the CRISPR-mediated technology (https://clinicaltrials.gov/).

**Status**	**Study title**	**Conditions**	**Intervention**	**Locations**	**Informations**
Active, not recruiting	β-Thalassemia major with autologous CD34+ hematopoietic progenitor cells transduced with TNS9.3.55 a lentiviral vector encoding the normal human ß-Globin Gene (NCT01639690)	Confirmed diagnosis of β-thalassemia Major	Autologous CD34+ cells transduced with TNS9.3.55	Memorial Sloan Kettering Cancer Center, New York, United States	The stem cells are collected from the patients and the abnormal genes are removed. The cells are treated to induce the normal hemoglobin production before being infused back to the patients.
Enrolling by invitation	A study evaluating the safety and efficacy of the BD211 drug product in β-thalassemia major participants (NCT05015920)	Hematologic diseases	BD211 drug product	920th hospital of joint logistics support force of people's liberation army of China kunming, Yunnan, China	The patient's autologous cells are enriched for CD34+ HSCs and undergo ex vivo transduction with lentiviral vector encoding βA-T87Q-globin to BD211 finished product, which is then infused intravenously into the patient.
Active, not recruiting	A safety and efficacy study evaluating CTX001 in subjects with transfusion-dependent β-thalassemia (NCT03655678)	β-thalassemia Thalassemia Genetic diseases, inborn (and 2 more...)	CTX001	Stanford University, Stanford, California, United States Ann & Robert Lurie Children's Hospital of Chicago, Chicago, Illinois, United States Columbia University, Manhattan, New York, United States (and 9 more...)	The study will evaluate the safety and efficacy of autologous CRISPR-Cas9 Modified CD34+ Human Hematopoietic Stem and Progenitor Cells (hHSPCs) using CTX001.
Enrolling by invitation	A long-term follow-up study in subjects who received CTX001 (NCT04208529)	β-thalassemia Thalassemia Sickle cell disease (and 4 more...)	CTX001	Columbia University Medical Center (21+ years), New York, United States Columbia University Medical Center, New York, United States St. Jude Children's Research Hospital Memphis, Tennessee, United States (and 8 more...)	This is an observational study to evaluate the long-term safety and efficacy of CTX001 in subjects who received CTX001 in Study CTX001-111 (NCT03655678) or VX21-CTX001-141 (transfusion-dependent β-thalassemia [TDT] studies) or Study CTX001-121 (NCT03745287) or VX21-CTX001-151 (severe sickle cell disease [SCD] studies; NCT05329649).
Not yet recruiting	Evaluation of safety and efficacy of CTX001 in pediatric participants with transfusion-dependent β-thalassemia (TDT) (NCT05356195)	β-thalassemia Thalassemia Genetic diseases, Inborn (and 2 more...)	CTX001	N/A	This study will evaluate the safety and efficacy of autologous CRISPR-Cas9 modified CD34+ human hematopoietic stem and progenitor cells (hHSPCs) (CTX001).
Active, not recruiting	Safety and efficacy evaluation of ET-01 transplantation in subjects with transfusion dependent β-thalassaemia (NCT04390971)	Transfusion dependent β-thalassaemia	ET-01	Institute of Hematology & Blood Diseases Hospital, Tianjin, China	This study evaluates the safety and Efficacy of ET-01 Transplantation in subjects with Transfusion Dependent β-Thalassaemia.
Active, not recruiting	A safety and efficacy study evaluating ET-01 in subjects with transfusion dependent β-thalassaemia (NCT04925206)	Transfusion dependent β-thalassaemia	ET-01	Nanfang Hospital of Southern Medical University, Guangzhou, Guangdong, China Guangzhou Women and Children's Medical Center, Guangzhou, Guangdong, China Shenzhen Children's Hospital, Shenzhen, Guangdong, China Institute of Hematology & Blood Diseases Hospital, Tianjin, China	This study will evaluate the safety and efficacy of autologous CRISPR-Cas9 Modified CD34+ Human Hematopoietic Stem and Progenitor Cells (hHSPCs) using ET-01.
Active, not recruiting	A study evaluating the efficacy and safety of the lentiglobin® BB305 drug product in participants with transfusion-dependent β-thalassemia (NCT03207009)	β-thalassemia	LentiGlobin BB305 drug product	UCSF Benioff Children's Hospital Oakland, Oakland, California, United States Ann & Robert H. Lurie Children's Hospital of Chicago, Chicago, Illinois, United States Children's Hospital of Philadelphia, Philadelphia, Pennsylvania, United States (and 6 more...)	This is a single-arm, multi-site, single-dose, Phase 3 study in approximately 18 participants less than or equal to (< =) 50 years of age with transfusion-dependent β-thalassemia (TDT), who have a β0/β0, β0/IVS-I-110, or IVS-I-110/IVS-I-110 genotype. The study will evaluate the efficacy and safety of autologous hematopoietic stem cell transplantation (HSCT) using LentiGlobin BB305 Drug Product.
Completed	A study evaluating the safety and efficacy of the lentiglobin bb305 drug product in β-thalassemia major participants (NCT01745120)	β-thalassemia Major	LentiGlobin BB305 Drug Product	Los Angeles, California, United States Oakland, California, United States Chicago, Illinois, United States (and 3 more...)	This study will evaluate the safety and efficacy of autologous hematopoietic stem cell transplantation (HSCT) using LentiGlobin BB305 Drug Product [autologous CD34+ hematopoietic stem cells transduced with LentiGlobin BB305 lentiviral vector encoding the human βA-T87Q-globin gene]
Completed	A study evaluating the safety and efficacy of lentiglobin BB305 drug product in β-thalassemia major (also referred to as transfusion-dependent β-thalassemia [TDT]) and sickle cell disease (NCT02151526)	β-thalassemia Major Sickle Cell Disease	LentiGlobin BB305 Drug Product	Paris, France	This study evaluates the safety, and efficacy study of the administration of LentiGlobin BB305 Drug Product to participants with either transfusion dependent beta-thalassemia (TDT) or sickle cell disease (SCD).
Not yet recruiting	Safety and efficacy evaluation of β-globin restored autologous hematopoietic stem cells in β-thalassemia major patients (NCT04592458)	β-thalassemia major	LentiHBBT87Q	Beijing Genomics Institute, Shenzhen, Guangdong, China	The patient's autologous hematopoietic stem cells will be collected and modified with LentiHBBT87Q system to restore the β-globin expression.
					The corrected autologous hematopoietic stem cells will be infused back to patients, and will be monitored the long-term safety and efficacy of the treatment for up to 13 years post-transplantation.
Active, not recruiting	Long-term follow-up of subjects treated with OTL-300 for transfusion dependent β-thalassemia study (TIGET-BTHAL) (NCT03275051)	β-thalassaemia	OTL-300	Ospedale San Raffaele - Telethon Institute for Gene Therapy (OSR-TIGET) Milan, Italy	OTL-300 is a gene therapy drug product consisting of autologous hematopoietic stem/progenitor cluster of differentiation (CD) 34+ cells genetically modified with a lentiviral vector (GLOBE) encoding the human beta globin gene. The TIGET-BTHAL is a phase I/II study evaluating safety and efficacy of OTL-300 in subjects with transfusion dependent beta-thalassemia for 2 years post gene-therapy.
Enrolling by invitation	β-globin restored autologous HSC in β-thalassemia major patients (NCT04205435)	β-thalassemia major	β-globin restored autologous HSC	Shanghai Bioraylaboratory Inc., Shanghai, China	This is a single center, single arm, open-label study to determine the safety and efficacy of β-globin restored autologous hematopoietic stem cells in β- thalassemia major patients with IVS-654 mutation. β-globin restored autologous hematopoietic stem cells will be manufactured using CRISPR/Cas9 gene editing system.
Recruiting	Safety and efficacy evaluation of γ-globin reactivated autologous hematopoietic stem cells (NCT04211480)	β-thalassemia Major	γ-globin reactivated autologous hematopoietic stem cells	Shanghai Bioray Laboratories Inc., Shanghai, China	This study aims to evaluate the safety and efficacy of the treatment with γ-globin reactivated autologous hematopoietic stem cells in subjects with β-thalassemia major. γ-globin reactivated autologous hematopoietic stem cells will be manufactured using CRISPR/Cas9 gene editing system.

## Conclusions

The accumulated research evidences to date demonstrate that genome editing technologies have made substantial contributions to the development of treatment options for a variety of human diseases. In the present review, we summarized the applications of different gene editing tools including ASOs, U7 snRNA, ZFNs, TALENs, and CRISPR/Cas9 systems. ASOs-driven correction of aberrant splicing is sequence and mutation specific, thus limited to certain cases of β-thalassemia. Nevertheless, some of the most common mutations that cause aberrant splicing are responsible for almost 90% of thalassemia cases worldwide (1). Hence, a small number of ASOs may be useful for treatment of a large majority of thalassemia patients. The lifelong therapeutic effect of U7 snRNA has captured the attention along with its compact size and less toxicity properties. The advancement of HDR-based DNA repair through ZFNs, TALENs, and CRISPR/Cas9-mediated double strand break has promised a potential approach to cure the β-thalassemia genetically. Nonetheless, despite the significant opportunities for therapy and translational research, as well as recent technological advancements, gene therapy still has some limitations, including design difficulties and costs associated with the use of ZFNs, TALENs, and CRISPR/Cas9, off target effects, low transfection efficiency, *in vivo* delivery-safety, and ethical concerns.

## Author Contributions

MJ and RB: conceptualization and writing—review and editing. NZ: writing—original draft preparation. WA, AM, RA, and SS: visualization. MJ, RB, and AM: supervision. MJ: funding acquisition. All authors have read and agreed to the published version of the manuscript.

## Funding

This work was supported by Fundamental Research Grant Scheme (FRGS/1/2018/SKK08/USM/02/6) to MJ from the Ministry of Higher Education, Malaysia.

## Conflict of Interest

The authors declare that the research was conducted in the absence of any commercial or financial relationships that could be construed as a potential conflict of interest.

## Publisher's Note

All claims expressed in this article are solely those of the authors and do not necessarily represent those of their affiliated organizations, or those of the publisher, the editors and the reviewers. Any product that may be evaluated in this article, or claim that may be made by its manufacturer, is not guaranteed or endorsed by the publisher.

## References

[1] Weatherall DJ. Thalassemia as a global health problem: recent progress toward its control in the developing countries. Ann N Y Acad Sci. (2010) 1202:17–23. 10.1111/j.1749-6632.2010.05546.x 20712767

[2] Cao A Galanello R. Beta-thalassemia. Genet Med. (2010) 12:61–76. 10.1097/GIM.0b013e3181cd68ed 20098328

[3] Mishra AK Tiwari A. Iron overload in beta thalassaemia major and intermedia patients. Maedica (Bucur). (2013) 8:328–32. 24790662 PMC3968466

[4] Matta BN Musallam KM Maakaron JE Koussa S Taher AT A killer revealed: 10-year experience with beta-thalassemia intermedia. Hematology. (2014) 19:196–8. 10.1179/1607845413Y.0000000120 24074485

[5] Borgna-Pignatti C Rugolotto S De Stefano P Zhao H Cappellini MD Del Vecchio GC. Survival and complications in patients with thalassemia major treated with transfusion and deferoxamine. Haematologica. (2004) 89:1187–93. 15477202

[6] Zurlo MG De Stefano P Borgna-Pignatti C Di Palma A Piga A Melevendi C. Survival and causes of death in thalassaemia major. Lancet. (1989) 2:27–30. 10.1016/S0140-6736(89)90264-X 2567801

[7] Asadov C Alimirzoeva Z Mammadova T Aliyeva G Gafarova S Mammadov J. β-thalassemia intermedia: a comprehensive overview and novel approaches. Int J Hematol. (2018) 108:5–21. 10.1007/s12185-018-2411-9 29380178

[8] Nienhuis AW Nathan DG. Pathophysiology and clinical manifestations of the β-thalassemias. Cold Spring Harb Perspect Med. (2012) 2:a011726. 10.1101/cshperspect.a011726 23209183 PMC3543079

[9] Huang C Qu Y Liu S Nie S Jiang H. Hematopoietic stem cell transplantation for thalassemia major using hla fully-matched and mismatched donor grafts. Transl Pediatr. (2021) 10:1552–65. 10.21037/tp-20-415 34295770 PMC8261584

[10] Giardini C Lucarelli G. Bone marrow transplantation for beta-thalassemia. Hematol/Oncol Clin North America. (1999) 13:1059–64, viii. 10.1016/S0889-8588(05)70109-X 10553261

[11] Mustafa M Qatawneh M Al Jazazi M Jarrah O Al Hazaimeh R Oudat R. Hematopoietic stem cell transplantation in thalassemia patients: a jordanian single centre experience. Mater Sociomed. (2020) 32:277–82. 10.5455/msm.2020.32.277-282 33628130 PMC7879431

[12] Merli P Ruggeri A Algeri M Li Pira G Ceglie G Gruppioni K. Clinical outcomes after allogeneic hematopoietic stem cell transplantation in patients with transfusion-dependent β-thalassemia treated at the bambino gesù children's hospital, Rome, Italy. Blood. (2019) 134:969. 10.1182/blood-2019-123440

[13] Moi P Sadelain M. Towards the genetic treatment of β-thalassemia: new disease models, new vectors, new cells. Haematologica. (2008) 93:325–30. 10.3324/haematol.12732 18310536

[14] Giardine B Borg J Viennas E Pavlidis C Moradkhani K Joly P. Updates of the hbvar database of human hemoglobin variants and thalassemia mutations. Nucleic Acids Res. (2014) 42:D1063–9. 10.1093/nar/gkt911 24137000 PMC3964999

[15] Wild BJ Green BN Cooper EK Lalloz MR Erten S Stephens AD. Rapid identification of hemoglobin variants by electrospray ionization mass spectrometry. Blood Cells Mol Dis. (2001) 27:691–704. 10.1006/bcmd.2001.0430 11482884

[16] Fucharoen S Weatherall DJ. The hemoglobin e thalassemias. Cold Spring Harb Perspect Med. (2012) 2:a011734. 10.1101/cshperspect.a011734 22908199 PMC3405827

[17] Thein SL. The molecular basis of β-thalassemia. Cold Spring Harb Perspect Med. (2013) 3:a011700. 10.1101/cshperspect.a011700 23637309 PMC3633182

[18] Olivieri NF Pakbaz Z Vichinsky E. Hb E/Beta-thalassaemia: a common & clinically diverse disorder. Indian J Med Res. (2011) 134:522–31. 22089616 PMC3237252

[19] Olivieri NF Pakbaz Z Vichinsky E. Hbe/β-thalassemia: basis of marked clinical diversity. Hematol Oncol Clin North Am. (2010) 24:1055–70. 10.1016/j.hoc.2010.08.008 21075280

[20] Corey DR Abrams JM. Morpholino antisense oligonucleotides: tools for investigating vertebrate development. Genome Biol. (2001) 2:1–3. 10.1186/gb-2001-2-5-reviews1015 11387041 PMC138935

[21] Nualkaew T Jearawiriyapaisarn N Hongeng S Fucharoen S Kole R Svasti S. Restoration of correct βivs2-654-globin MRNA splicing and hba production by engineered U7 snrna in β-thalassaemia/Hbe erythroid cells. Sci Rep. (2019) 9:7672. 10.1038/s41598-019-43964-3 31113996 PMC6529457

[22] Lacerra G Sierakowska H Carestia C Fucharoen S Summerton J Weller D. Restoration of hemoglobin a synthesis in erythroid cells from peripheral blood of thalassemic patients. Proc Natl Acad Sci U S A. (2000) 97:9591–6. 10.1073/pnas.97.17.9591 10944225 PMC16909

[23] Suwanmanee T Sierakowska H Lacerra G Svasti S Kirby S Walsh CE. Restoration of human beta-globin gene expression in murine and human ivs2-654 thalassemic erythroid cells by free uptake of antisense oligonucleotides. Mol Pharmacol. (2002) 62:545–53. 10.1124/mol.62.3.545 12181431

[24] Svasti S Suwanmanee T Fucharoen S Moulton HM Nelson MH Maeda N. Rna repair restores hemoglobin expression in ivs2–654 thalassemic mice. Proc Nat Acad Sci. (2009) 106:1205. 10.1073/pnas.0812436106 19164558 PMC2633555

[25] Suwanmanee T Sierakowska H Fucharoen S Kole R. Repair of a splicing defect in erythroid cells from patients with beta-Thalassemia/Hbe Disorder. Mol Ther. (2002) 6:718–26. 10.1006/mthe.2002.0805 12498768

[26] El-Beshlawy A Mostafa A Youssry I Gabr H Mansour IM El-Tablawy M. Correction of aberrant pre-mrna splicing by antisense oligonucleotides in beta-thalassemia egyptian patients with ivsi-110 mutation. J Pediatr Hematol Oncol. (2008) 30:281–4. 10.1097/MPH.0b013e3181639afe 18391696

[27] Dong A Ghiaccio V Motta I Guo S Peralta R Freier SM. 2'-O-Methoxyethyl splice-switching oligos correct splicing from ivs2-745 beta-thalassemia patient cells restoring hba production and chain rebalance. Haematologica. (2019) 106:1433–42. 10.3324/haematol.2019.226852 32439726 PMC8094087

[28] Gadgil A Raczynska KD. U7 Snrna: a tool for gene therapy. J Gene Med. (2021) 23:e3321. 10.1002/jgm.3321 33590603 PMC8243935

[29] Vacek MM Ma H Gemignani F Lacerra G Kafri T Kole R. High-level expression of hemoglobin a in human thalassemic erythroid progenitor cells following lentiviral vector delivery of an antisense snrna. Blood. (2003) 101:104–11. 10.1182/blood-2002-06-1869 12393543

[30] Phanthong P Borwornpinyo S Kitiyanant N Jearawiriyapaisarn N Nuntakarn L Saetan J. Enhancement of β-globin gene expression in thalassemic ivs2-654 induced pluripotent stem cell-derived erythroid cells by modified u7 snrna. Stem Cells Transl Med. (2017) 6:1059–69. 10.1002/sctm.16-0121 28213976 PMC5442829

[31] d'Arqom A Nualkaew T Jearawiriyapaisarn N Kole R Svasti S. Engineered U7 small nuclear Rna restores correct β-globin pre-mrna splicing in mouse β(Ivs2-654)-thalassemic erythroid progenitor cells. Human Gene Ther. (2021) 32:473–80. 10.1089/hum.2020.145 32977730

[32] Preedagasamzin S Nualkaew T Pongrujikorn T Jinawath N Kole R Fucharoen S. Engineered U7 snrna mediates sustained splicing correction in erythroid cells from β-thalassemia/Hbe patients. Biochem Biophys Res Commun. (2018) 499:86–92. 10.1016/j.bbrc.2018.03.102 29550480

[33] Magrin E Miccio A Cavazzana M. Lentiviral and genome-editing strategies for the treatment of β-hemoglobinopathies. Blood. (2019) 134:1203–13. 10.1182/blood.2019000949 31467062

[34] Ernst MPT Broeders M Herrero-Hernandez P Oussoren E van der Ploeg AT Pijnappel W. Ready for repair? Gene editing enters the clinic for the treatment of human disease. Molec Ther Methods Clin Develop. (2020) 18:532–57. 10.1016/j.omtm.2020.06.022 32775490 PMC7393410

[35] Hoban MD Cost GJ Mendel MC Romero Z Kaufman ML Joglekar AV. Correction of the sickle cell disease mutation in human hematopoietic stem/progenitor cells. Blood. (2015) 125:2597–604. 10.1182/blood-2014-12-615948 25733580 PMC4408287

[36] Genovese P Schiroli G Escobar G Tomaso TD Firrito C Calabria A. Targeted genome editing in human repopulating haematopoietic stem cells. Nature. (2014) 510:235–40. 10.1038/nature13420 24870228 PMC4082311

[37] Wang J Exline CM DeClercq JJ Llewellyn GN Hayward SB Li PW. Homology-driven genome editing in hematopoietic stem and progenitor cells using zfn mrna and aav6 donors. Nat Biotechnol. (2015) 33:1256–63. 10.1038/nbt.3408 26551060 PMC4842001

[38] De Ravin SS Reik A Liu PQ Li L Wu X Su L. Targeted gene addition in human Cd34(+) hematopoietic cells for correction of x-linked chronic granulomatous disease. Nat Biotechnol. (2016) 34:424–9. 10.1038/nbt.3513 26950749 PMC4824656

[39] Brusson M Miccio A. Genome editing approaches to β-hemoglobinopathies. Prog Mol Biol Transl Sci. (2021) 182:153–83. 10.1016/bs.pmbts.2021.01.025 34175041

[40] Smith AR Schiller GJ Vercellotti GM Kwiatkowski JL Krishnamurti L Esrick EB. Preliminary results of a phase 1/2 clinical study of zinc finger nuclease-mediated editing of bcl11a in autologous hematopoietic stem cells for transfusion-dependent beta thalassemia. Blood. (2019) 134:3544. 10.1182/blood-2019-125743

[41] Ma N Liao B Zhang H Wang L Shan Y Xue Y. Transcription activator-like effector nuclease (talen)-mediated gene correction in integration-free β-thalassemia induced pluripotent stem cells. J Biol Chem. (2013) 288:34671–9. 10.1074/jbc.M113.496174 24155235 PMC3843079

[42] Patsali P Turchiano G Papasavva P Romito M Loucari CC Stephanou C. Correction of Ivs I-110(G>a) β-thalassemia by crispr/cas-and talen-mediated disruption of aberrant regulatory elements in human hematopoietic stem and progenitor cells. Haematologica. (2019) 104:e497–501. 10.3324/haematol.2018.215178 31004018 PMC6821606

[43] Xu P Tong Y. Liu X-z, Wang T-t, Cheng L, Wang B-y, et al. Both talens and crispr/Cas9 directly target the Hbb Ivs2-654 (C > T) mutation in beta-thalassemia-derived Ipscs. Sci Rep. (2015) 5:12065. 10.1038/srep12065 26156589 PMC4496796

[44] Fang Y Cheng Y Lu D Gong X Yang G Gong Z. Treatment of β(654) -thalassaemia by talens in a mouse model. Cell Prolif. (2018) 51:e12491. 10.1111/cpr.12491 30070404 PMC6528953

[45] Song B Fan Y He W Zhu D Niu X Wang D. Improved hematopoietic differentiation efficiency of gene-corrected beta-thalassemia induced pluripotent stem cells by Crispr/Cas9 system. Stem Cells Dev. (2015) 24:1053–65. 10.1089/scd.2014.0347 25517294

[46] Xiong Z Xie Y Yang Y Xue Y Wang D Lin S. Efficient gene correction of an aberrant splice site in β-thalassaemia ipscs by crispr/cas9 and single-strand oligodeoxynucleotides. J Cell Mol Med. (2019) 23:8046–57. 10.1111/jcmm.14669 31631510 PMC6850948

[47] Xu S Luk K Yao Q Shen AH Zeng J Wu Y. Editing aberrant splice sites efficiently restores β-globin expression in β-thalassemia. Blood. (2019) 133:2255–62. 10.1182/blood-2019-01-895094 30704988 PMC6533605

[48] Gabr H El Ghamrawy MK Almaeen AH Abdelhafiz AS Hassan AOS El Sissy MH. Crispr-mediated gene modification of hematopoietic stem cells with beta-thalassemia ivs-1-110 mutation. Stem Cell Res Ther. (2020) 11:390. 10.1186/s13287-020-01876-4 32912325 PMC7488347

[49] Xie F Ye L Chang JC Beyer AI Wang J Muench MO. Seamless gene correction of β-thalassemia mutations in patient-specific ipscs using crispr/cas9 and piggybac. Genome Res. (2014) 24:1526–33. 10.1101/gr.173427.114 25096406 PMC4158758

[50] Li J Zhou Z Sun HX Ouyang W Dong G Liu T. Transcriptome analyses of β-thalassemia −28(a>G) mutation using isogenic cell models generated by crispr/cas9 and asymmetric single-stranded oligodeoxynucleotides (Assodns). Front Genet. (2020) 11:577053. 10.3389/fgene.2020.577053 33193694 PMC7580707

[51] Niu X He W Song B Ou Z Fan D Chen Y. Combining single strand oligodeoxynucleotides and crispr/cas9 to correct gene mutations in β-thalassemia-induced pluripotent stem cells. J Biol Chem. (2016) 291:16576–85. 10.1074/jbc.M116.719237 27288406 PMC4974373

[52] Liu Y Yang Y Kang X Lin B Yu Q Song B. One-step biallelic and scarless correction of a β-thalassemia mutation in patient-specific ipscs without drug selection. Molec Ther Nucl Acids. (2017) 6:57–67. 10.1016/j.omtn.2016.11.010 28325300 PMC5363452

[53] Wattanapanitch M Damkham N Potirat P Trakarnsanga K Janan M. One-step genetic correction of hemoglobin e/beta-thalassemia patient-derived Ipscs by the Crispr/Cas9 system. Stem Cell Res Ther. (2018) 9:46. 10.1186/s13287-018-0779-3 29482624 PMC5828150

[54] Cosenza LC Gasparello J Romanini N Zurlo M Zuccato C Gambari R. Efficient crispr-cas9-based genome editing of β-globin gene on erythroid cells from homozygous β(0)39-thalassemia patients. Molec Ther Methods Clin Develop. (2021) 21:507–23. 10.1016/j.omtm.2021.03.025 33997100 PMC8091488

[55] Cai L Bai H Mahairaki V Gao Y He C Wen Y. A universal approach to correct various Hbb gene mutations in human stem cells for gene therapy of beta-thalassemia and sickle cell disease. Stem Cells Transl Med. (2018) 7:87–97. 10.1002/sctm.17-0066 29164808 PMC5746148

[56] Ye L Wang J Tan Y Beyer AI Xie F Muench MO. Genome editing using crispr-cas9 to create the hpfh genotype in hspcs: an approach for treating sickle cell disease and β-thalassemia. Proc Natl Acad Sci U S A. (2016) 113:10661–5. 10.1073/pnas.1612075113 27601644 PMC5035856

[57] Pavani G Fabiano A Laurent M Amor F Cantelli E Chalumeau A. Correction of β-thalassemia by crispr/cas9 editing of the α-globin locus in human hematopoietic stem cells. Blood advances. (2021) 5:1137–53. 10.1182/bloodadvances.2020001996 33635334 PMC7948300

[58] Cromer MK Vaidyanathan S Ryan DE Curry B Lucas AB Camarena J. Global transcriptional response to crispr/cas9-aav6-based genome editing in Cd34+ hematopoietic stem and progenitor cells. Molecular Therapy. (2018) 26:2431–42. 10.1016/j.ymthe.2018.06.002 30005866 PMC6171165

